# Ribosomal DNA methylation in human and mouse oocytes increases with age

**DOI:** 10.18632/aging.203891

**Published:** 2022-02-14

**Authors:** Ramya Potabattula, Tom Trapphoff, Marcus Dittrich, Kinga Fic, Grazyna E. Ptak, Stefan Dieterle, Thomas Haaf

**Affiliations:** 1 Institute of Human Genetics, Julius Maximilians University, Würzburg, Germany; 2 Fertility Center, Dortmund, Germany; 3 Department of Bioinformatics, Julius Maximilians University, Würzburg, Germany; 4 Malopolska Centre of Biotechnology (MCB), Jagiellonian University, Krakow, Poland; 5 Division of Reproductive Medicine and Infertility, Department of Obstetrics and Gynecology, Witten/Herdecke University, Dortmund, Germany

**Keywords:** bisulfite pyrosequencing, human and mouse GV oocytes, ovarian aging, ribosomal DNA methylation, single oocyte analysis

## Abstract

An age-dependent increase in ribosomal DNA (rDNA) methylation has been observed across a broad spectrum of somatic tissues and the male mammalian germline. Bisulfite pyrosequencing (BPS) was used to determine the methylation levels of the rDNA core promoter and the rDNA upstream control element (UCE) along with two oppositely genomically imprinted control genes (*PEG3* and *GTL2*) in individual human germinal vesicle (GV) oocytes from 90 consenting women undergoing fertility treatment because of male infertility. Apart from a few (4%) oocytes with single imprinting defects (in either *PEG3* or *GTL2*), the analyzed GV oocytes displayed correct imprinting patterns. In 95 GV oocytes from 42 younger women (26-32 years), the mean methylation levels of the rDNA core promoter and UCE were 7.4±4.0% and 9.3±6.1%, respectively. In 79 GV oocytes from 48 older women (33-39 years), methylation levels increased to 9.3±5.3% (*P* = 0.014) and 11.6±7.4% (*P* = 0.039), respectively. An age-related increase in oocyte rDNA methylation was also observed in 123 mouse GV oocytes from 29 4-16-months-old animals. Similar to the continuously mitotically dividing male germline, ovarian aging is associated with a gain of rDNA methylation in meiotically arrested oocytes. Oocytes from the same woman can exhibit varying rDNA methylation levels and, by extrapolation, different epigenetic ages.

## INTRODUCTION

In developed countries, the trend towards delayed parenthood has been constantly increasing. For economic, social, political, and cultural reasons, many parents postpone their wish for children beyond the optimal biological age [1]. This has led to an ever-increasing demand for assisted reproductive technologies and prenatal diagnostic testing. For decades, it is well known that the oocyte aneuploidy rate and, consequently, the risk for fertility problems, miscarriages, and children with Down syndrome (and other chromosome disorders) increases with maternal age. Major underlying factors of reduced fertility at advanced maternal age are the decline in ovarian reserve [2] and the increased rate of chromosomal non-disjunction due to the prolonged meiotic arrest [3, 4]. In addition, aggregation of meiotic double-stranded breaks [5], loss of homologous recombination proteins [6] and cohesins [7], accumulation of oxidative stress [8], and a severe bottle neck in mitochondrial DNA segregation [9] may contribute to the maternal age-related medical problems. Transcriptome analyses revealed numerous age-related changes in oocyte gene expression including genes for cell cycle regulation, energy production, and other critical pathways [10–13].

In the male germline, the number of spermatogonial cell divisions increases from 35 at puberty to >800 at 50 years [14]. During each cell division, not only the DNA sequence but also its epigenetic modifications must be copied to the daughter cells. Considering that the error rate during this copying process is at least one order of magnitude higher for epigenetic information than for genetic information [15], the sperm epigenome can be expected to acquire 10-100 times more age-related epimutations than DNA sequence mutations. Accumulating evidence suggests an association between advanced paternal age and DNA methylation changes in the sperm epigenome and also in the next generation [16–18]. In contrast to the well-studied effects of advanced paternal age on the sperm epigenome, little is known about the possible impact of maternal age on the epigenome of the non-dividing meiotically arrested oocyte that is transcriptionally silenced at the end of the oocyte growth phase. During growth, the oocyte accumulates large numbers of ribosomes that are contributed to the preimplantation embryo for gene expression before zygotic gene activation [12, 19]. Methylcytosine staining revealed an age-related decrease in global DNA methylation in oocytes of aged female mice [20], whereas a review of more recent studies reported a trend towards increased global DNA methylation in aged germinal vesicle (GV) oocytes [21].

Repetitive elements like ribosomal DNA (rDNA) comprise over two-thirds of the human genome [22]. Recently, we have shown that different repeat DNA families, including (peri)centromeric satellite DNAs, interspersed retrotransposons, and rDNA gain methylation in the aging male mammalian germ line [23]. The human genome is endowed with several hundred (315±104) rDNA transcription units, each containing more than 1,500 CpG sites [24]. Promoter methylation of the tandemly arrayed transcription units on the acrocentric short arms leads to epigenetic rDNA silencing [25], whereas gene body methylation may enhance rDNA transcription [26]. In turn, rDNA transcription affects ribosome biogenesis, overall protein synthesis, and essentially each cellular process. The nucleolus is the central organelle for protein production during oocyte growth which can be expected to have an impact on the quality of the oocyte and the resulting preimplantation embryo [12, 27].

Interestingly, the correlation between biological aging and rDNA methylation has been conserved across a broad spectrum of somatic tissues and species [28–31]. In contrast to other epigenetic clocks which are built on the methylation levels of highly selected CpGs scattered throughout the genome [32–34], rDNA methylation may reflect functional changes in nucleolar biology during aging and in age-related conditions [35, 36]. Although age-associated rDNA methylation has been proposed as a universal age predictor [31], ovarian aging has not been studied so far. Here, we have analyzed rDNA methylation of individual human oocytes from ovum pick-ups for assisted reproduction as well as mouse GV oocytes.

## RESULTS

### Methylation analysis of imprinted genes in human oocytes

Imprinted genes show a parent-of-origin-specific expression of either the paternal or the maternal allele [37]. They are generally used as controls to exclude somatic cell contamination in epigenetic studies on mature germ cells. Here, we established a multiplex PCR assay including the oppositely imprinted control regions of *PEG3* (maternally methylated) and *GTL2* (paternally methylated) as well as two target regions (core promoter and UCE) of the rDNA transcription unit.

**Figure 1:**
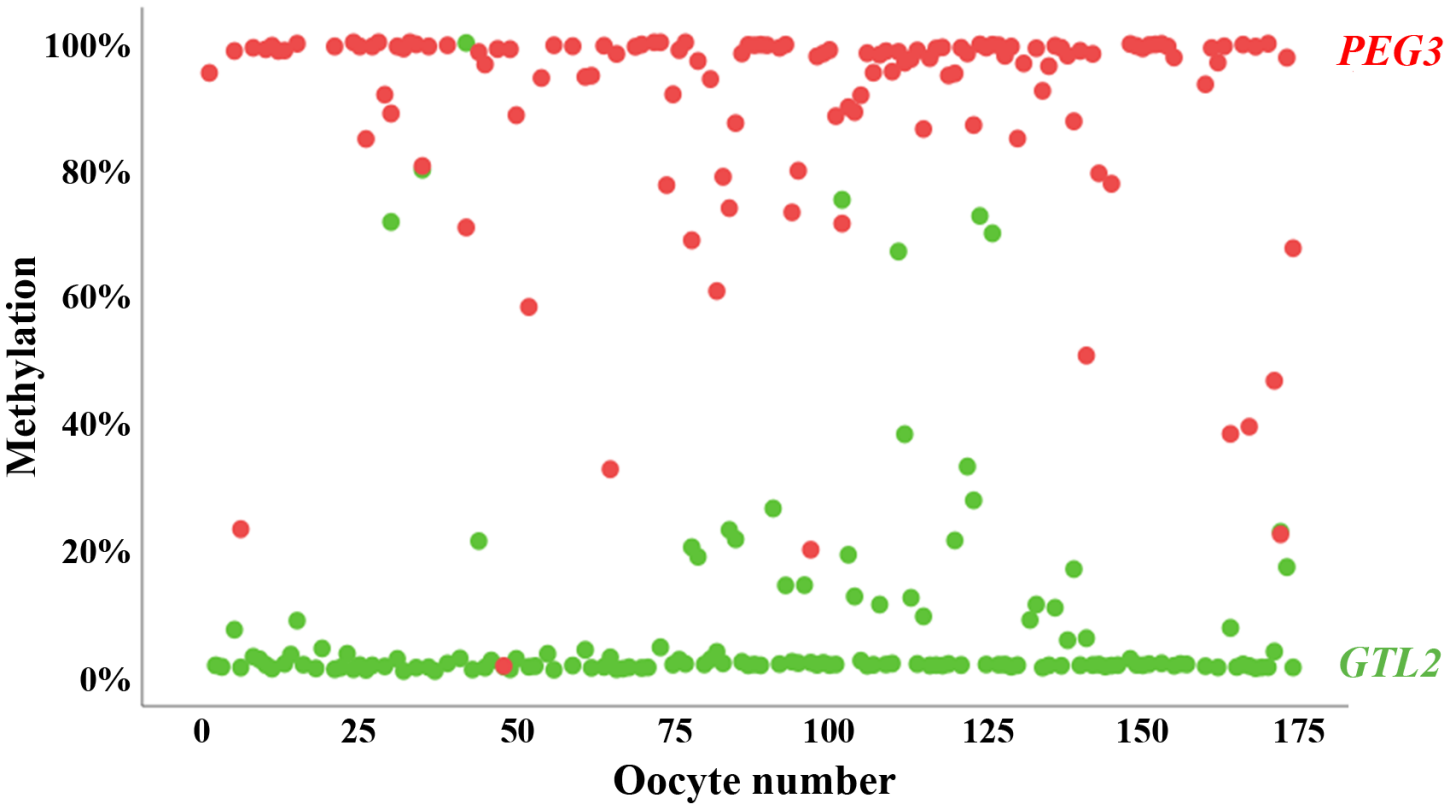
**Methylation of oppositely imprinted regions in individual human oocytes.** Mean methylation of *PEG3* (red dots) and *GTL2* (green dots) in 174 individual human GV oocytes included in this study. Only oocytes with correct oocyte methylation of at least one of the two analyzed imprinted genes were taken for further analysis. The vast majority (88%) of oocytes display correct methylation for both controls.

Most (153 of 174; 88%) oocytes showed >80% methylation for the maternally imprinted (paternally expressed) *PEG3* and <20% methylation for the paternally imprinted (maternally expressed) *GTL2* gene (Figure 1). A small percentage (7 of 174; 4%) displayed an abnormal methylation pattern of one imprinted gene but not of the other. For 14 of 174 (8%) oocytes, we did not obtain PCR products of *PEG3* and/or *GTL2* for methylation analysis. Taken together, we can exclude somatic cell contamination and inferior oocyte quality in our cohort. The mean (± standard deviation, SD) methylation in the analyzed oocytes was 90±19% (median 98%) for *PEG3* and 7.6±17% (median 1.0%) for *GTL2*.

### Age-related increase in oocyte rDNA methylation in humans and mice

**Figure 2:**
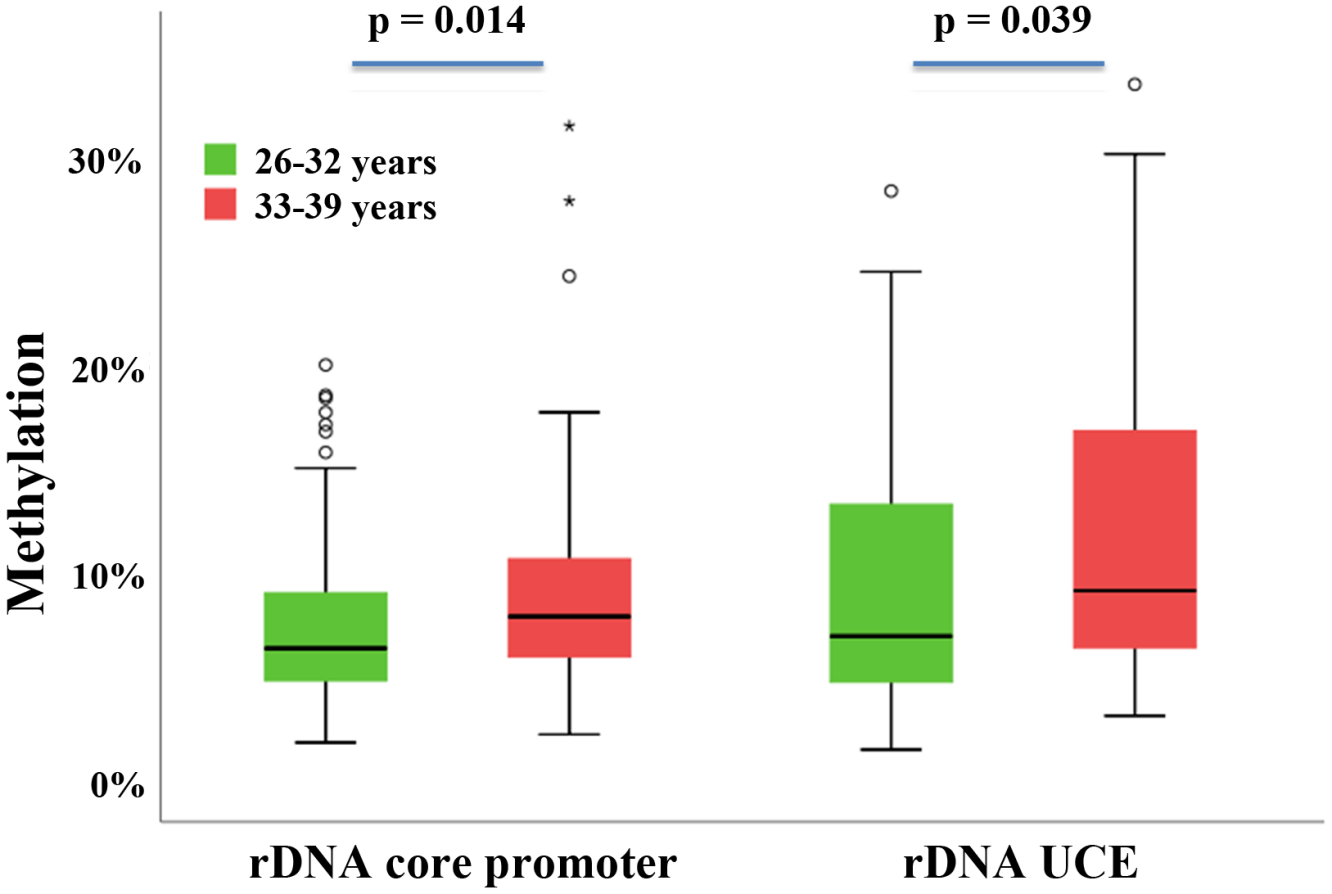
**rDNA methylation difference between oocytes from younger versus older women.** Box plots showing the rDNA core promoter and rDNA UCE methylation in younger women (26-32 years; *N* = 42) and older women (33-39 years; *N* = 48). The median is represented by a horizontal line. The bottom of the box indicates the 25^th^ percentile, the top the 75^th^ percentile. Outliers are shown as circles and extreme outliers as stars. The methylation levels in the older group are higher compared to the younger group.

A positive correlation between male age and sperm rDNA methylation has been conserved in the male germline of different mammalian species [23]. To test whether a comparable age effect exists in the female germline, bisulfite pyrosequencing (BPS) of the rDNA core promoter and UCE was performed using individual human GV oocytes. Ninety-five oocytes were obtained from 42 “younger” women between 26 and 32 years and 79 oocytes from 48 “older” women from 33 to 39 years (Supplementary Figure 1). To test for the methylation differences between the two age groups, linear mixed-effect models were used accounting for the repeated measures of multiple oocytes per woman. For the rDNA promoter amplicon, the mean (±SD) methylation was 7.4±4.0% (median 6.4%) for the younger women and 9.3±5.3% (median 7.9%) for the older women (Figure 2). For the UCE, the mean methylation was 9.3±6.1% (median 6.9%) in the younger group and 11.6±7.4% (median 9.2%) in the older group. The between-group difference was significant for both the core promoter (*P* = 0.014) and UCE (*P* = 0.039). Both the rDNA core promoter (Spearman's rho = 0.23; *P* = 0.039) and UCE (rho = 0.24; *P* = 0.038) methylation were positively correlated with donor age (Supplementary Figure 2). Moreover, there was a significant positive correlation (Spearman’s rho = 0.42, *P* <0.0001) between the methylation levels of the rDNA core promoter and the rDNA UCE (Supplementary Figure 3A). Our results suggest that human ovarian aging is associated with increasing oocyte methylation of the rDNA promoter and UCE. In contrast, the body mass index (BMI) did not show a significant or trend correlation with rDNA methylation.

**Figure 3:**
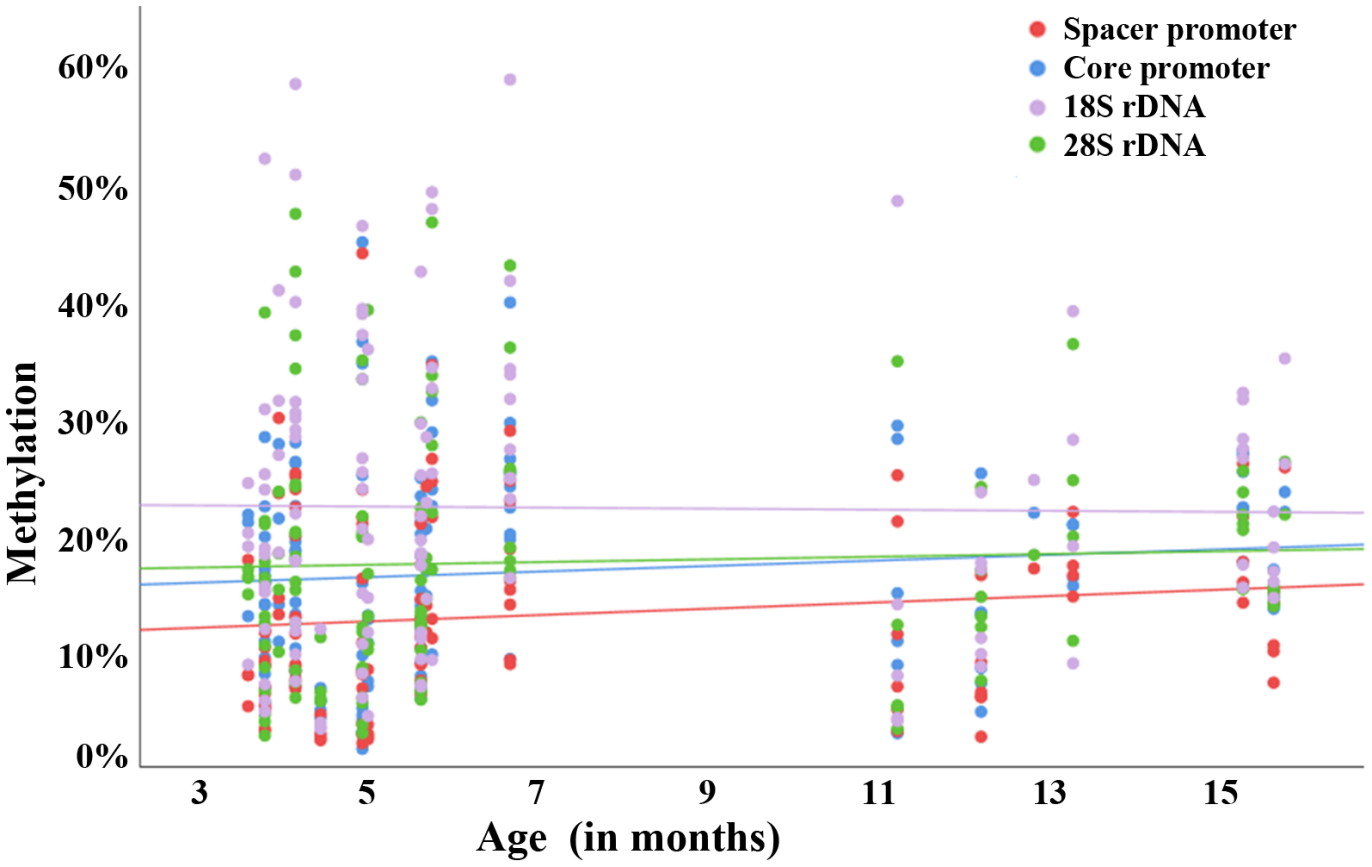
**Raw correlation between rDNA methylation and donor age in individual mouse oocytes.** Scatter plot showing a positive correlation between donor age (x-axis in months) and methylation (y-axis in %) of the rDNA spacer promoter (red dots), core promoter (blue dots), 18S rDNA (mauve dots), and 28S rDNA (green dots). Altogether, 123 GV oocytes from 29 4-16-months-old mice were analyzed. Each dot represents an individual oocyte.

To uncover whether this maternal age effect has been evolutionarily conserved, oocyte methylation of the rDNA (spacer and core) promoter regions and gene body (18S and 28S rDNA) was analyzed in the aging mouse model. Forty-one GV oocytes were obtained from ten 4-months-old, 51 from ten 5-7-months-old, and 31 from nine 11-16-months-old females. Correlation of raw oocyte methylation with age was significant or trend significant for the spacer promoter (Spearman's rho = 0.27; *P* = 0.004), core promoter (rho = 0.20; *P* = 0.03), and 28S rDNA (rho = 0.17; *P* = 0.06), whereas no significant changes were observed for 18S rDNA (Figure 3). Mean methylation of all oocytes per animal showed similar correlations (rho = 0.32 for the spacer promoter, 0.20 for the core promoter, 0.08 for 18S rDNA, and 0.19 for 28S rDNA), albeit not reaching significance, probably due to the smaller number of donors (Supplementary Figure 4). The oocyte methylation levels of any two analyzed mouse rDNA regions were significantly (rho = 0.43-0.83; *P* <0.001) correlated with each other (Supplementary Figure 3B).

### rDNA methylation variation between germ cells of the same donor

**Figure 4:**
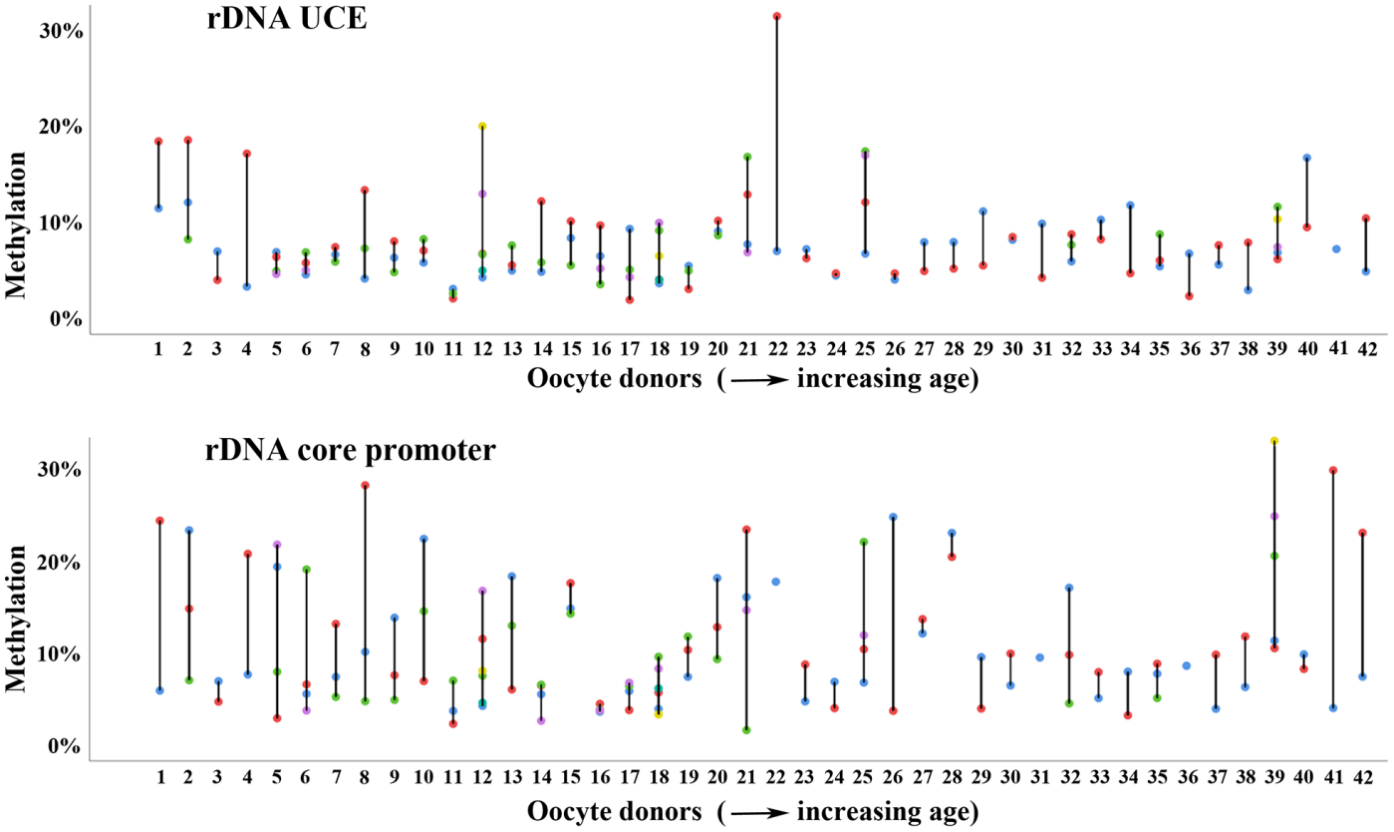
**Methylation variation of multiple oocytes from the same woman.** Methylation variation of the rDNA UCE (upper panel) and core promoter (lower panel) in individual (color-coded) oocytes from the same woman. Women 1-42 are arranged with increasing age (from 26 to 39 years) on the x-axis. Most women, i.e. numbers 3 and 23 show similar methylation values in different oocytes and rDNA amplicons. Some women, i.e. 12 and 21 display enormous methylation variation between oocytes.

Forty-two women contributed multiple (up to 6) oocytes to this study (Supplementary Figure 1). Interestingly, for some donors (i.e. 2, 4, 8, 12, 21, 25, and 42), both rDNA UCE and core promoter methylation varied dramatically (in the order of 10-20%) between different oocytes from the same retrieval (Figure 4). It seems plausible that overall methylation variation increases with the number of oocytes obtained from the same women. However, huge methylation variation was also observed between 2 or 3 oocytes, i.e. from donors 1, 4, and 8, and relatively little variation between 4 to 6 oocytes, i.e. from donors 17 and 18. The majority (24 of 42; 57%) of women displayed similar methylation levels (<5% variation) in different oocytes and rDNA amplicons. Some graphic examples are women 3, 19, 23, 24, 27, and 33 (Figure 4). Considering that the technical variation of bisulfite pyrosequencing is 1-2% for genomic DNA samples with millions of DNA molecules, <5% methylation variation between individual oocytes of the same women demonstrates the reliability of our measurements. Similar methylation variation between individual oocytes (up to 9) from the same donor were observed in the aging mouse model (Supplementary Figure 5).

## DISCUSSION

### Age-dependent changes in nucleolar structure, gene expression and methylation in oocytes

The 45S pre-rRNA transcript of the rDNA transcription unit is spliced into 5.8S, 18S, and 28S rRNAs, which are assembled in the ribosomal subunits of the nucleolus. Alterations in nucleolar biology are thought to play a functional role in the aging process [35, 36]. In both humans [38] and mice [39, 40], functional nuclear architecture has been used to classify GV oocytes into two main types: A larger group with “surrounded nucleolus (SN)” which may be able to fully sustain early embryo development and a smaller group with “not surrounded nucleolus (NSN)” which usually leads to developmental arrest. The rDNA in SN oocytes with a dense ring of chromatin around the nucleolus is transcriptionally silenced, whereas the uncondensed chromatin in NSN oocytes is still transcriptionally active. Oocytes from reproductively old mice have a higher percentage of nucleoli with giant fibrillary centers, indicative of increased rDNA transcription, and more ribosomes in the cytoplasm than oocytes from younger animals [12]. Collectively, these results suggest that alterations in chromatin compaction and transcriptional silencing during the oocyte growth phase impact oocyte quality. The age-associated decline of female fertility appears to be primarily due to reduced oocyte quality rather the quantity [27, 41].

An age-dependent increase in rDNA methylation has been observed in widely different somatic tissues and sperm samples of humans and other mammalian species [28–31]. An evolutionarily conserved rDNA methylation clock appears to operate in similar ways in the soma [31] and male germline [23]. This study shows for the first time that in humans maternal aging is associated with an overall statistically significant gain of rDNA UCE and core promoter methylation in meiotically arrested oocytes. Weak correlations between donor age and oocyte rDNA methylation of spacer promoter, core promoter and 28S rDNA were also observed in the mouse model. In contrast to previous studies on age-related rDNA methylation changes in sperm, here we analyzed individual human oocytes. BPS of human semen samples representing millions of individual sperm revealed 0.33% (*P* < 0.0001) increments in rDNA methylation per year for both the core promoter and the UCE [23]. A similar age effect with 0.18% (*P* = 0.075) and 0.28% (*P* = 0.064) increments per year, respectively, was seen in human oocytes. It is noteworthy that the age effect on rDNA methylation in somatic tissues such as blood (0.06% increments per year; *P* < 0.01) is one order of magnitude smaller than in the germline [23]. The methylation levels of all analyzed rDNA regions (UCE and core promoter in humans; spacer and core promoter, 18S and 28S rDNA in the mouse) significantly correlated with each other. Thus, similar to the aging male mammalian germline, the entire rDNA transcription unit in oocytes may be susceptible to aging. In contrast to promoter regions, gene body methylation promotes transcription of rDNA [26].

In somatic cells and spermatogonial stem cells, methylation changes could be due to errors in the maintenance of DNA methylation patterns and epigenetic drift. Since oocytes in aging women do not divide, active de novo methylation must occur at the rDNA UCE and promoter regions. Methylation of rDNA in somatic cells critically depends on activity of the maintenance DNA methyltransferase DNMT1 and the de novo DNA methyltransferase DNMT3B [42]. Recently, it has been shown that to some extent DNMT1 also catalyzes de novo methylation of specific repeats [43]. The upstream non-transcribed spacers might facilitate 5’ to 3’ spreading of methylation, an event commonly seen in age-related diseases [44]. In oocytes of aged female mice, both *DNMT* transcript and protein levels appear to be reduced [20, 45] and cytoplasm-to-nucleus trafficking of DNMTs during oocyte maturation may be compromised [21, 46].

### Epigenetic clocks in germ cells

Recently, several epigenetic clocks were built on highly selected CpGs with age-related methylation changes to estimate the biological age of cattle and human oocytes [47]. Overall, there was little overlap between age-related CpGs in oocytes and blood and also a much lower number of significant CpGs in oocytes. In contrast to blood, where cells are continually renewed, oocytes already stop dividing in the fetal germline and from then on, their number is constantly declining. In this light, the female germline may be the first "organ" to fail during an organism's lifespan. Epigenetic clocks based on a subset of age-related CpGs suggest that immature bovine oocytes start at an older epigenetic age and age more slowly than somatic tissues [47]. The relationship (cause, consequence, or mere bystander) of clock CpGs to the aging process remains unclear [33]. It is generally assumed that the calculated DNA methylation age represents a surrogate marker that tracks the cumulative work done by an epigenetic maintenance system [32] and/or an age-dependent decay of the methylation landscape [33]. In contrast to these clock CpGs, the age-related rDNA methylation changes in both human sperm [23] and oocytes are higher than in somatic tissue (blood) and thought to be functionally relevant.

Some women displayed dramatic rDNA methylation variation (in the order of 10-20%) between individual oocytes from the same retrieval. Single-oocyte methylome analysis revealed that global CpG methylation patterns have been largely established in the immature GV oocytes and remain stable to the mature MII stage, whereas non-CpG sites methylation undergoes remodeling through the final stages of maturation [48]. In this study, immature GV oocytes that failed to resume final maturation were obtained from large antral follicles after ovarian stimulation and hCG priming. Studies to induce *in vitro* maturation to metaphase II in immature GV oocytes suggest that immature oocytes from stimulated cycles may represent a rather heterogeneous group [49, 50]. Although we cannot exclude the formal possibility that the observed rDNA methylation variation is due to different developmental stages of the studied oocytes, it is tempting to speculate that oocytes with extreme methylation values (>20%) and methylation variation are compromised in their developmental competence.

In the mouse model, the majority of oocytes of older females displayed reduced complexity and increased variation of the transcriptome, associated with reduced developmental potential compared to oocytes from younger animals. Interestingly, a limited number of "old" oocytes exhibited a "young-like" epigenome [13]. Single oocyte methylomes from aged mice displayed decreased global CpG methylation (of single-copy genes), whereas our results show increased rDNA methylation in aging mouse and human oocytes. This age-related gain in rDNA methylation appears to be conserved in both the male [23] and female mammalian germline, which may be considered a good indicator of functional significance. Consistent with the analysis of mouse oocyte transcriptomes, a proportion of oocytes from older women were endowed with rDNA methylation levels similar to those from younger oocytes. Considering that rDNA methylation of both oocytes and sperm increases with age, germ cells with lower methylation values may be epigenetically younger and, by extrapolation, have a higher chance to establish a pregnancy. In somatic tissues, the DNA methylation age is increasingly used as a biomarker for biological aging, lifetime prediction, and rejuvenating interventions [33, 34].

### Possible impact of age-related rDNA methylation changes to the next generation

CpG methylation is a mechanism for regulating rDNA transcription and nucleolar activity. Promoter methylation inhibits assembly of the transcription initiation process and, consequently rDNA transcription and expression [25]. In contrast, gene body methylation prevents binding of repressive histone marks to maintain transcription [26]. Advanced parental age is associated with increased methylation of rDNA UCE and core promoter in both oocyte and sperm, which may affect nucleolar biology of the resulting embryos. After fertilization, the rapidly dividing embryos are highly dependent on efficient ribosome biogenesis and protein synthesis. Overall, the rDNA methylation of germ cells increases about 3% with every 10 years of parental age. A significant number of germ cells (from both younger and older women) display extreme rDNA methylation values of >20%. Impaired ribosome biogenesis due to rDNA hypermethylation may negatively influence embryo development. Until human embryonic genome activation at the eight-cell stage [51], early development entirely depends on maternally inherited RNAs and proteins. Subsequent embryonic development requires paternal and maternal rDNA transcription and highly efficient ribosome biogenesis. Despite enormous advances in embryo culture techniques, approximately half of human IVF/ICSI embryos arrest development before the blastocyst stage [52]. Accumulating evidence suggests that the rDNA epigenetic states during early development are not only important for nucleolar activity but also for higher-order functional organization of the embryonic genome [53]. Moreover, oocytes have to provide large quantities of ribosomes to the zygote at fertilization for translational activation of maternal RNAs before zygotic gene activation [19]. Therefore, alterations in oocyte rDNA promoter methylation in oocytes may adversely affect developmental potential.

### Limitations

This study is based on immature human GV oocytes which were not suitable for IVF/ICSI. It is only possible to a limited extent to extrapolate findings from oocytes of women undergoing ICSI to the general population. Only few oocytes displayed imprinting defects in either *GTL2* or *PEG3*, which are generally thought to be due to ovulation induction and other stressors during oocyte development [54]. In the human female germline, maternal methylation imprints are established during later stages of oocyte growth and for some genes may not be completed until shortly before pronuclear fusion [37]. However, consistent with the mouse model [13], the vast majority of human oocytes from both "younger" and "older" females displayed correct oocyte imprinting patterns. This also largely excludes somatic cell contamination and technical artefacts. For ethical reasons, it was not possible to collect mature human oocytes. Moreover, we only had oocytes from women with a limited age range (from 26 to 39 years). Although the analyzed oocytes appear to show the same age-dependent increase of rDNA methylation as sperm, the statistical significance for a maternal age effect is not quite as strong as for the paternal one.

BPS of single cells is challenging, because sodium bisulfite degrades most of the DNA, whereas too mild reaction conditions lead to incomplete conversion of cytosine. In our analysis of two imprinted genes, 88% of individual oocytes displayed the expected methylation patterns for both *PEG3* (median 98%) and *GTL2* (median 1%). Allele drop-out was observed in only 8% of cells. Compared to single-copy genes, single-cell BPS of several hundred copies of rDNA is much more robust. We estimate that the technical error is <5%, compared to about 1-2% for genomic DNA samples. In our opinion, methylation variation of 10-20% between individual oocytes reflects biological differences rather than experimental noise.

The observed age-related rDNA methylation changes in oocytes were small (in the order of several percentage points) and comparable to those in sperm [23]. Because of the enormous variation of DNA methylation patterns among individuals and among individual cells, there was considerable overlap in rDNA methylation values between oocytes from the older and the younger group. However, small effect size and large methylation variation do not necessarily exclude functional importance. Embryo development is a highly coordinated process, depending on numerous factors. Consistent with a multifactorial model, multiple changes of small effect size in the oocyte and/or sperm, exceeding a critical threshold, may cause developmental arrest.

In our study, maternal BMI did not reveal a significant effect on oocyte rDNA methylation. Smoking was not considered as a confounding factor, however the number of smokers in the analyzed cohort was low (<10%). Although we cannot exclude the possibility that our findings are restricted to oocytes retrieved after ovarian stimulation for human infertility treatment, increasing rDNA methylation is thought to be a hallmark of ovarian aging.

## MATERIALS AND METHODS

### Study samples

Immature human GV oocytes were obtained by oocyte retrieval from large antral follicles after ovarian stimulation and hCG priming from women undergoing ICSI treatment due to male infertility. Women with endometriosis, polycystic ovary syndrome, cancer, and an anti-Mullerian hormone concentration <1 ng/ml were excluded. Altogether, 174 human GV oocytes were collected between 2018 and 2020 from 90 women and pseudonymized at the Fertility Center, Dortmund. Supplementary Figure 1 shows the distribution of age, body mass index (BMI), and the number of oocytes per donor. To avoid somatic cell contamination, oocytes were freed from the granulosa cells, rinsed in phosphate-buffered saline (PBS), and stored at -80° C until further investigation.

Mouse oocytes were isolated from the ovaries of 4-16-months-old C57BL/6 mice after cervical dislocation. Animals were maintained in a temperature- and light-controlled room (22° C and 12-h light-dark cycle) and were provided with food and water ad libitum. The removed ovaries were kept in PBS at room temperature and immediately processed. GV oocytes were isolated by rupturing the ovary with the use of a 30-gauge needle in M2 medium to release cumulus-oocyte complexes. Denudation was performed by mechanical removal of cumulus cells with a small-bore pipette. Then, the oocytes were washed three times in PBS and snap-frozen in 3 μl volume of PBS in 200 μl PCR tubes. Altogether, 123 immature GV oocytes were collected from 29 4-16-months-old mice between 2020 and 2021 at the Malopolska Center of Biotechnology, Krakow. Frozen oocytes were stored in a -80° C freezer until further analysis.

DNA from individual human and mouse oocytes was extracted and bisulfite converted using the EZ DNA Methylation Direct Kit (Zymo Research Corporation, Irvine, CA, USA), which is particularly suited for small amounts of DNA. Briefly, 10 μl of 2x digestion buffer and 1 μl of proteinase K (20 μg/μl) were added to the tube containing a single oocyte. After incubating for 20 min at 50° C, 130 μl of bisulfite conversion mix was added to each sample. The conversion reaction was performed in a thermal cycler at 98° C for 8 min and 64° C for 3.5 h. Bisulfite-treated DNA was cleaned with a spin column following the manufacturer’s recommendations and finally eluted in 10 μl of elution buffer. The bisulfite conversion rate is estimated to be >99% and the DNA recovery rate approximately 80%.

### Bisulfite pyrosequencing

PCR and pyrosequencing primers were designed with the Pyrosequencing Assay Design Software (Qiagen, Hilden, Germany). Multiplex PCR for the rDNA core promoter and UCE as well as the germline imprinting control regions of *PEG3* and *GTL2* was performed with single human oocytes. In the mouse, multiplex PCR of single oocytes was performed for the rDNA core promoter, spacer promoter, 18S, and 28S rDNA. The 25 μl reaction mixture contained 2.5 μl 10x PCR buffer with MgCl_2_, 0.5 μl (10 mM dNTPs) nucleotide mixture, 0.2 μl (5 U/μl) FastStart Taq DNA polymerase (Roche Diagnostics, Mannheim, Germany), 1.25 μl (10 pmol/ml) of forward and reverse outer primers (Supplementary Table 1) and 10 μl of bisulfite-converted DNA as a template. Amplifications were carried out with an initial denaturation step at 95° C for 5 min, 35 cycles of 95° C for 30 sec, 60° C (for human oocytes) or 58° C (for mouse) for 30 sec, and 72° C for 45 sec, and a final extension step at 72° C for 5 min.

Nested singleplex PCRs for each of the 8 studied amplicons were carried out using 2 μl of the first-round multiplex PCR product as a template. The 25 μl reaction mixture consisted of 2.5 μl 10x PCR buffer with MgCl_2_, 0.5 μl nucleotide mix, 0.2 μl FastStart Taq DNA polymerase, and 1.25 μl of forward and reverse inner primers (Supplementary Table 1). Cycling conditions were as follows: 95° C for 5 min, 35 cycles of 95° C for 30 sec, 60° C for 30 sec, and 72° C for 45 sec, and a final extension step at 72° C for 5 min. For *PEG3*, a 35-cycle reaction with an annealing temperature of 57° C was performed. Pyrosequencing was done using the PyroMark Gold Q96 CDT reagent kit and Pyro Q-CpG software on the PyroMark Q96 MD system (Qiagen). Unmethylated and fully methylated DNA standards (Qiagen) were used as controls in each pyrosequencing run.

### Statistical analysis

To compare groups with a similar age range and sample size in humans, two groups were evaluated: oocytes of women from 26 to 32 years were considered as “younger” and oocytes of women from 33 to 39 years as “older”. Both descriptive and inferential statistical analyses were performed using IBM SPSS version 28. Comparison of the methylation differences between groups was performed using linear mixed-effect models as implemented in the library (‘nlme’) of the computational statistics software R (version 3.6.3). The methylation values were log-transformed and regressed against the age groups as a categorical factor including “donor” as a random factor in the model to account for within-subject correlation of the repeated measures from multiple oocytes from the same woman. Spearman’s correlations were used for correlating the methylation levels between the analyzed rDNA amplicons (two in humans and four in mice). For correlation analysis between methylation of a given amplicon and donor age, the measurements of different oocytes were aggregated for each donor by average. A *P* value of < 0.05 was considered statistically significant throughout the analysis.

### Study approval

The study on human oocytes was approved by the ethics committee at Witten/Herdecke University (no. 240/2017 to S.D.). Written informed consent was obtained from all the participating women. Mouse ovaries were collected according to the guidelines of European Community Regulation 86/609 and the Polish Governmental Act on the protection of animals used for scientific or educational purposes (Dz.U. 2015 poz. 266).

## Supplementary Material

Supplementary Figures

Supplementary Table 1
