# Supplementary material for: Ribosomal DNA methylation in human and mouse oocytes increases with age
Source: Aging (Albany NY). 2022 Feb 14;14(3):1214–32. doi: 10.18632/aging.203891 (PMC8876901; doi:10.18632/aging.203891)
Supplement: Supplementary Figures [file aging-14-203891-s001.pdf]

SUPPLEMENTARY FIGURES

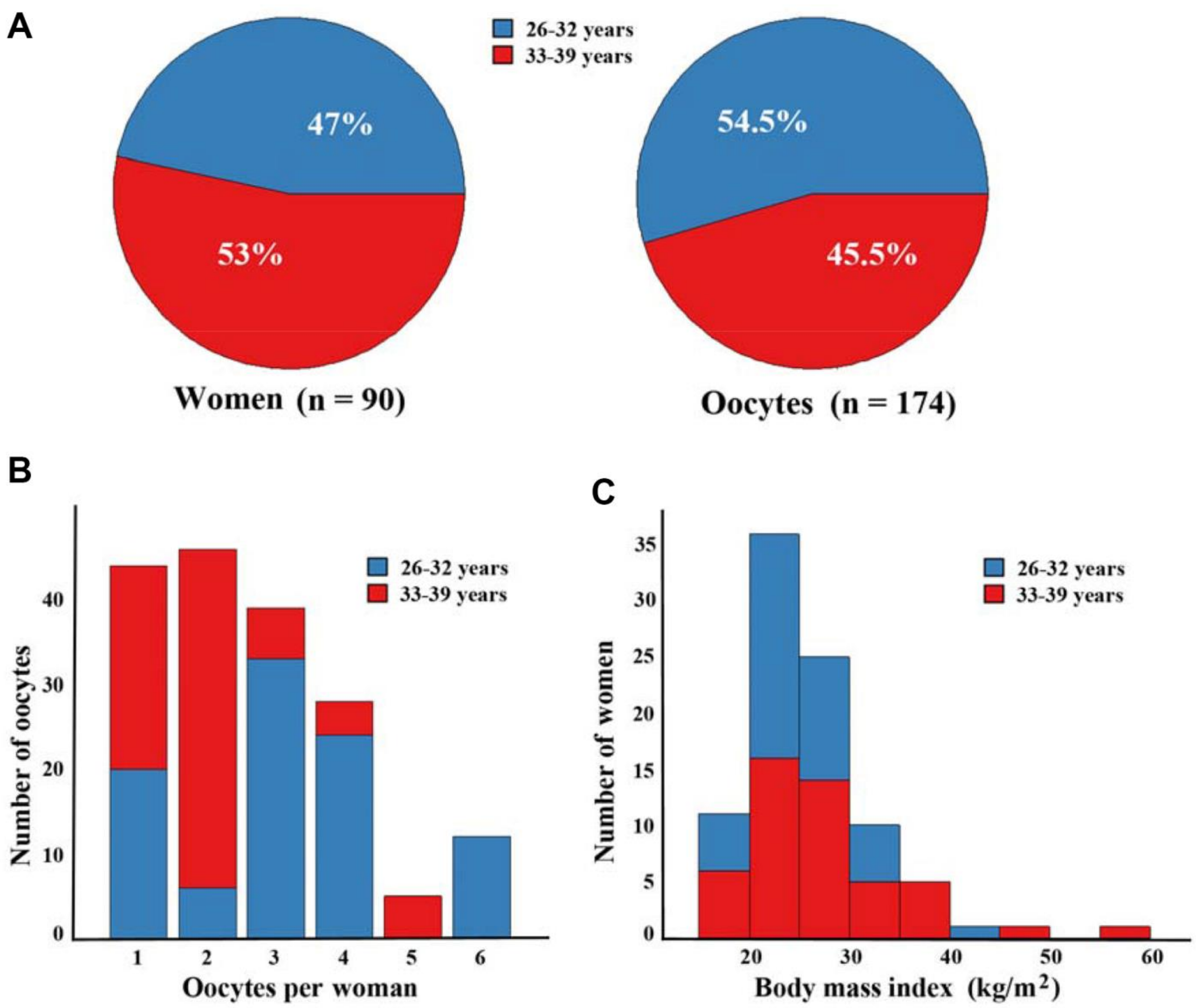

Supplementary Figure 1. Age, BMI, and number of oocytes per women. (**A**) Percentage of donors and oocytes from younger (26–32 years) and older women (33–39 years). (**B**) Distribution of oocyte number per women in younger and older women. (**C**) Distribution of maternal BMI in younger and older women.

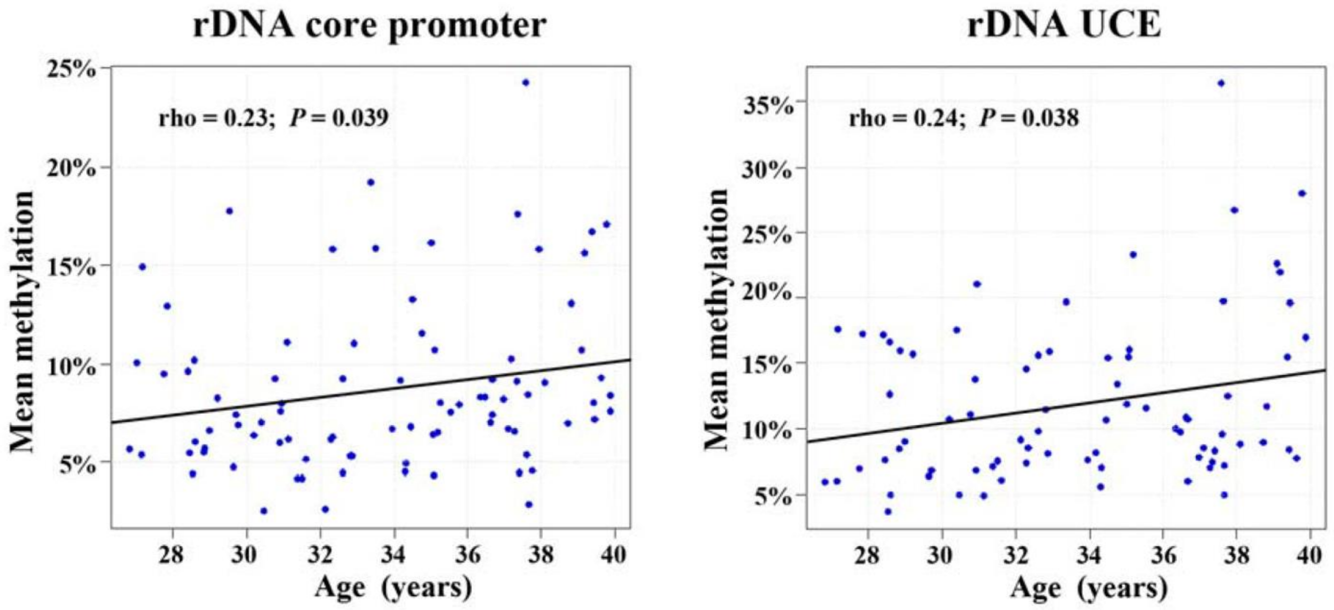

Supplementary Figure 2. Correlation between mean methylation (of different oocytes from the same women) and maternal age for the rDNA core promoter and UCE. Scatter plots showing a significant positive correlation between maternal age (x-axis in years) and mean methylation (y-axis in %) of the rDNA core promoter and UCE. Altogether, 174 oocytes from 90 women (blue dots) were analyzed. The measurements of different oocytes were aggregated for each donor by average.

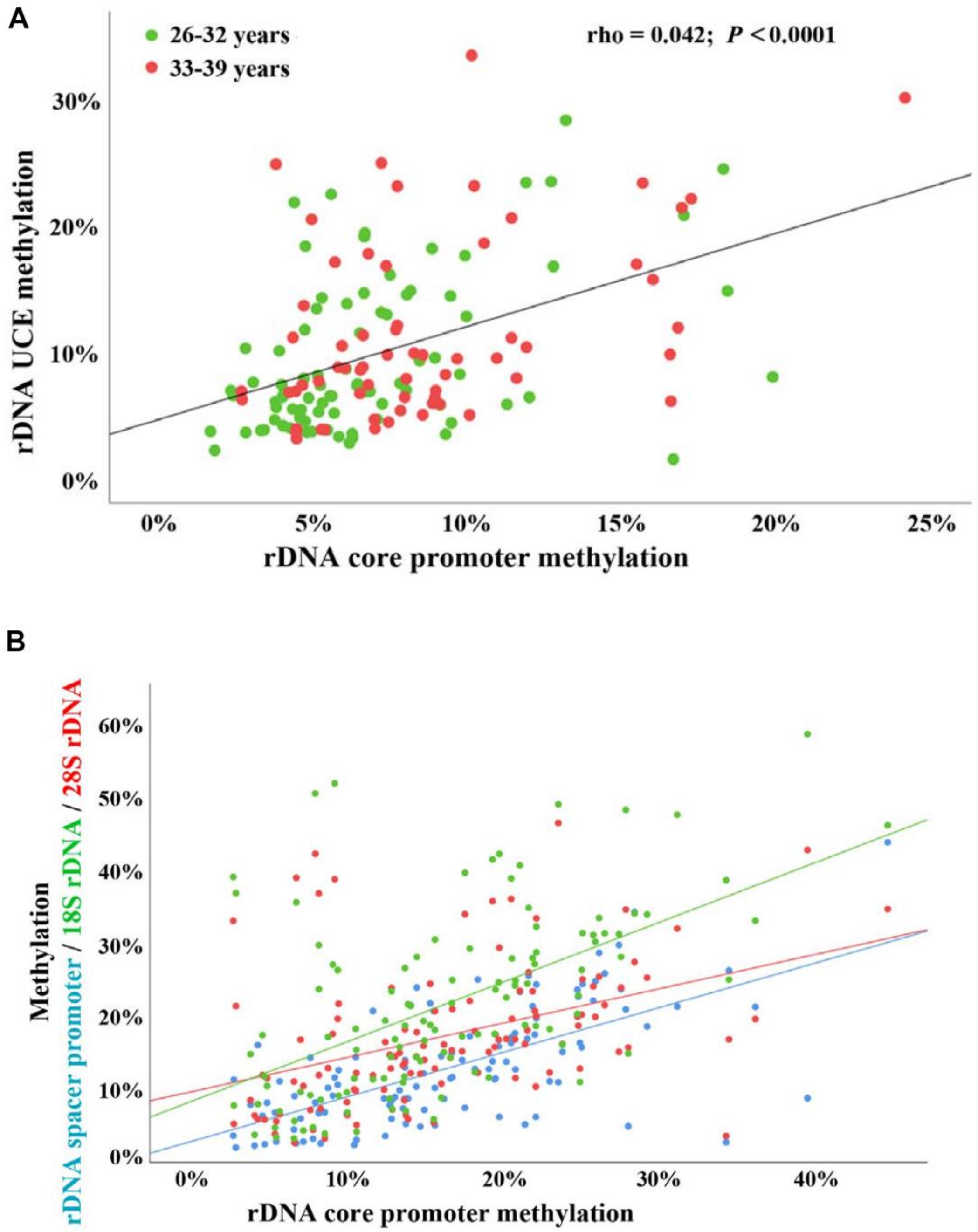

		18S rDNA	28S rDNA	Spacer promoter
Spearman's rho	Core promoter	0.53	0.48	0.65
P value		<0.001	<0.001	<0.001
Spearman's rho	Spacer promoter	0.43	0.43	
P value		<0.001	<0.001	
Spearman's rho	28S rDNA	0.83		
P value		<0.001		

Supplementary Figure 3. (**A**) Correlation between human rDNA core promoter and UCE methylation. Scatter plot showing a significant positive correlation between the methylation of the rDNA UCE (y-axis in %) and the rDNA core promoter (x-axis in %) in individual human GV oocytes. The oocytes from younger women (26-32 years) are represented by green dots and those from older women (33-39 years) by red dots. (**B**) Correlation between the methylation levels of different mouse rDNA amplicons (spacer promoter, core promoter, 18S rDNA, and 28S rDNA). Scatter plot showing significant positive correlations (see table) between methylation of the rDNA spacer promoter (blue dots), 18S rDNA (green dots), and 28S rDNA (red dots) (y-axis in %), respectively, and the rDNA core promoter (x-axis in %) in individual mouse GV oocytes.

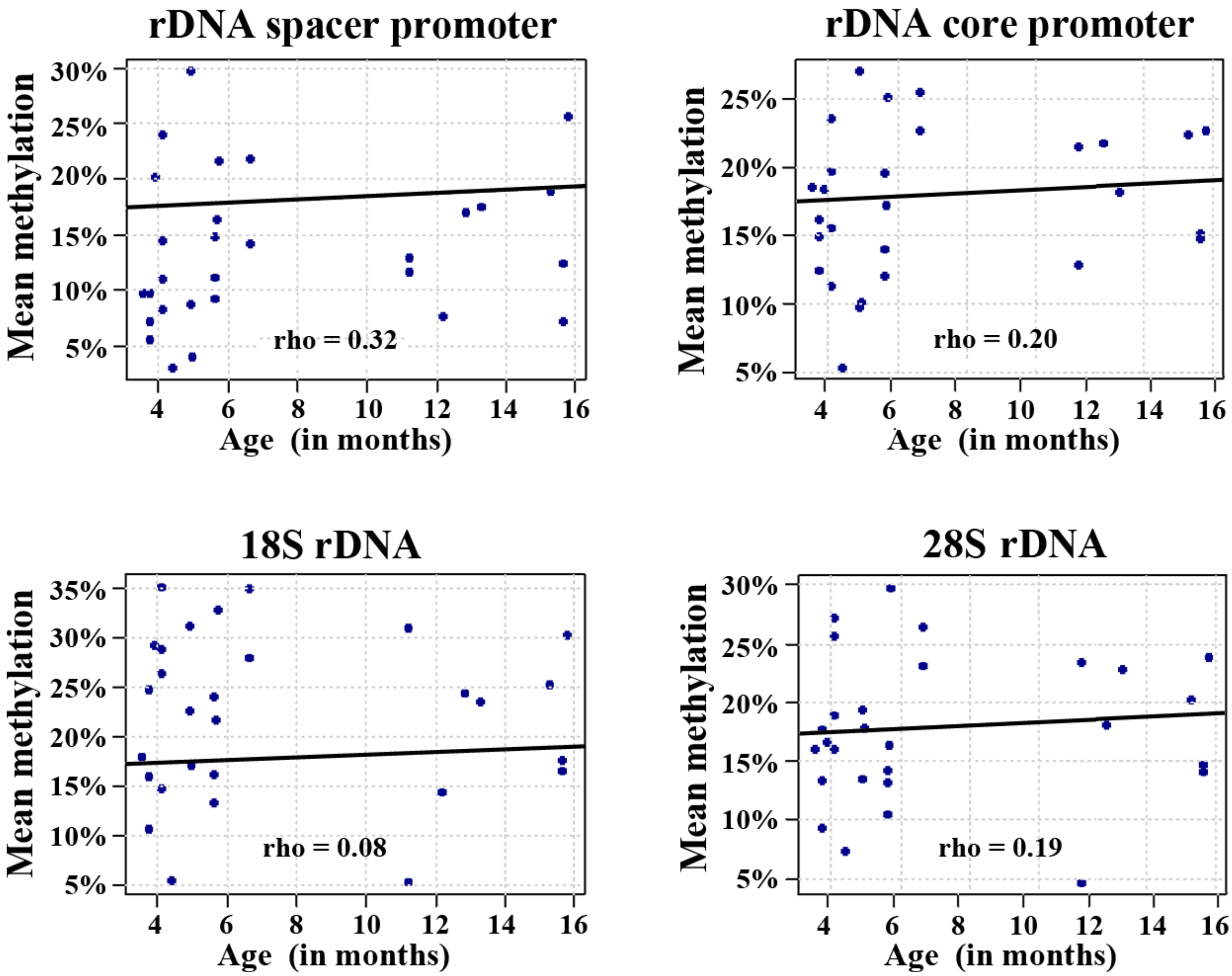

Supplementary Figure 4. Correlation between mean methylation (of different oocytes from the same animal) and donor age for the rDNA spacer promoter, core promoter, 18S and 28S rDNA. Scatter plots showing a positive correlation between donor age (x-axis in months) and mean methylation (y-axis in %) of the rDNA spacer promoter, core promoter, 18S and 28S rDNA. Altogether, 123 oocytes from 29 animals (blue dots) were analyzed. The measurements of different oocytes were aggregated for each donor by average.

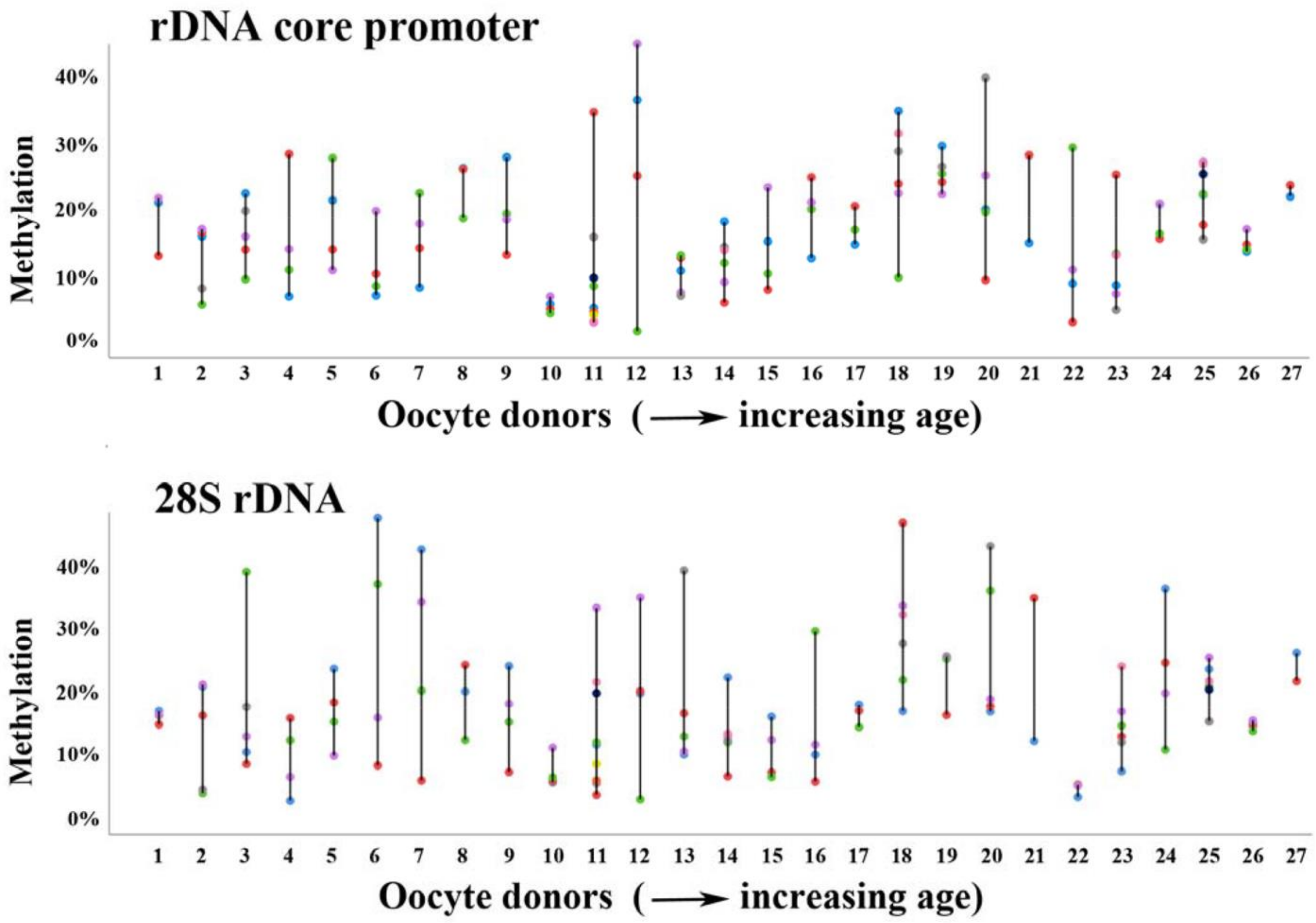

Supplementary Figure 5. Methylation variation of multiple mouse oocytes from the same donor. Methylation variation of the rDNA core promoter (upper panel) and 28S rDNA (lower panel) between individual (color-coded) oocytes from the same donor.
