# Supplementary material for: Ribosomal DNA methylation in human and mouse oocytes increases with age
Source: Aging (Albany NY). 2022 Feb 14;14(3):1214–32. doi: 10.18632/aging.203891 (PMC8876901; doi:10.18632/aging.203891)
Supplement: Supplementary Table 1 [file aging-14-203891-s002.pdf]

SUPPLEMENTARY TABLE

Supplementary Table 1. Primers for multiplex (outer) and singleplex (inner) PCR and pyrosequencing of single human and mouse oocytes.

Multiplex assay for single human oocytes				
**Target region**	**Primer**	**Sequence (5'-3')[a]**	**CpG number**	**Annealing temp:**
Human rDNA upstream control element[b]	Forward outer	GTGTTTTGGGGTTGATTAGAGG		60º C
	Reverse outer	ATCACCRATAAACCAAAACCCC		
	Forward inner	GTGTTTTTGGGTTGATTAGAGG		60º C
	Reverse inner	*CATCCCAAAACCCAACCTCTCC		
	Sequencing	GGTTGATTAGAGGGTT	5	
Human rDNA core promoterc	Forward outer	GGTAGTTGTYGAGGGAGGGG		60º C
	Reverse outer	AAAAAAACRTCCCCAACCTCC		
	Forward inner	GTTTTYGTTGTGAGTTAGGTAGAGTTT		60º C
	Reverse inner	*AAAAAAACRTCCCCAACCTCC		
	Sequencing	GGTTTATGTGGGGGAGAGGTTGT	9	
Human *GTL2* (paternally imprinted)	Forward outer	GAATTATAGGGAATGATGGTGTA		60º C
	Reverse outer	CCAAAATACTAACTACTCCTTAAACA		
	Forward inner	AGGGTTAGGAAGTTTAGTAGGTTA		60º C
	Reverse inner	*ACTACTCCTTAAACAAAAAAACACATAAT		
	Sequencing	GTAGTAAATTAAAGTGTATTAGAGA	5	
Human *PEG3* (maternally imprinted)	Forward outer	GGTTGTTGATTGGTTAGTATAG		60º C
	Reverse outer	CACTCACCTCACCTCAATAC		
	Forward inner	GGTGTAGAAGTTTGGGTAGTTG		57º C
	Reverse inner	*CTCACCTCACCTCAATACTAC		
	Sequencing	TGTTTATTTTGGGTTGGT	3	

Multiplex assay for single mouse oocytes				
**Target region**	**Primer**	**Sequence (5'-3')[a]**	**CpG number**	**Annealing temp:**
Mouse rDNA spacer promoter[d]	Forward outer	GTGTTTAATTGTGTTTGTTTT		58º C
	Reverse outer	CTCCTATATCACCAACCTAAAAAACCT		
	Forward inner	GGAGAAGTGGTGGGTGGG		60º C
	Reverse inner	*CTCCTATATCACCAACCTAAAAAACCT		
	Sequencing 1	AGTGAGTGAATGTGG	4	
	Sequencing 2	ATTGGTTTGTATGGTTGA	3	
Mouse rDNA core promoter[d]	Forward outer	GGAGGAAAGTGATAGGTTATAGAGAAT		58º C
	Reverse outer	TCCAAAAACCCTCTCTATCCC		
	Forward inner	TTGGGGAGGTGGTTTAAAAATGA		60º C
	Reverse inner	*CCTCCAAAAACCCTCTCTAT		
	Sequencing 1	GAGGTGGTTTAAAAATGAT	2	
	Sequencing 2	GGATTTTAAAGGAATAATTGGT	3	
Mouse 18S rDNA	Forward outer	TTAATTTTTTAGAGGGATAAGTGG		58º C
	Reverse outer	AAAACCTCACTAAACCATC		

	Forward inner	AGGTTTGTGATGTTTTTAGATGT		60° C
	Reverse inner	*AAAACCTCACTAAACCATC		
	Sequencing 1	TTTGTGATGTTTTTAGATGTT	4	
	Sequencing 2	ATTAAGTTTTTGTTTTTTGTATATA	4	
	Forward outer	TGGAGTAGAAGGGTAAAAGTT		58° C
	Reverse outer	CAACCAAACACATACACCAAATATCT		
Mouse 28S rDNA	Forward inner	GGTTTTAAGTAGGAGGTGTTAGAAAAG		60° C
	Reverse inner	*CAACCAAACACATACACCAAATATCT		
	Sequencing 1	GGATAATTGGTTTGTGG	7	
	Sequencing 2	GTTGGATTGTTTATTTATTAATAGG	3	

[a]Primers indicated by a star are biotinylated at the 5' end.
[b]Primers adopted from: Raval A, Sridhar KJ, Patel S, Turnbull BB, Greenberg PL, Mitchell BS. Reduced rRNA expression and increased rDNA promoter methylation in CD34+ cells of patients with myelodysplastic syndromes. Blood. 2012;120:4812-8.
[c]Primers adopted from: Teschler S, Gotthardt J, Dammann G, Dammann RH. Aberrant DNA methylation of rDNA and PRIMA1 in borderline personality disorder. Int J Mol Sci. 2016;17:E67.
[d]Primers adopted from: Shiao YH, Leighty RM, Wang C, Ge X, Crawford EB, Spurrier JM, McCann SD, Fields JR, Fornwald L, Riffle L, Driver C, Kasprzak KS, Quiñones OA, et al. Ontogeny-driven rDNA rearrangement, methylation, and transcription, and paternal influence. PloS One. 2012;6:e22266.
